# Supplementary material for: Design of High Affinity Binders to Convex Protein Target Sites
Source: bioRxiv. 2024 May 2:2024.05.01.592114. Preprint. [Version 1] doi: 10.1101/2024.05.01.592114 (PMC11092582; doi:10.1101/2024.05.01.592114)
Supplement: Supplement 1 [file media-1.pdf]

Supplementary Information

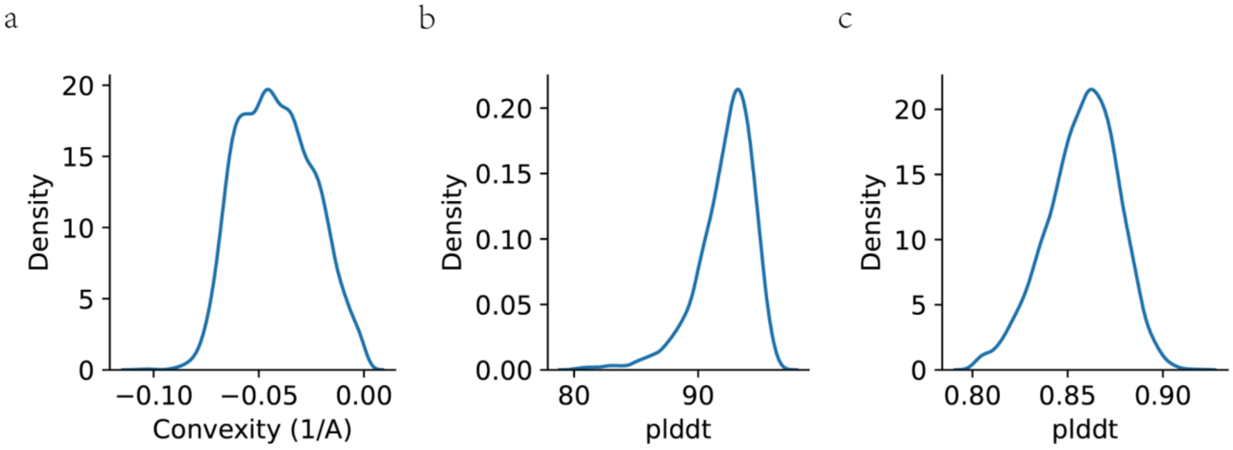

Figure S1. Computational Characterization of 5HCS scaffolds library. a, Distribution of curvatures of the 5HCS library. **b,** Distribution of pLDDT predicted by Alphafold2[1]. **c,** Distribution of plDDT predicted by DeepAccNet[2].

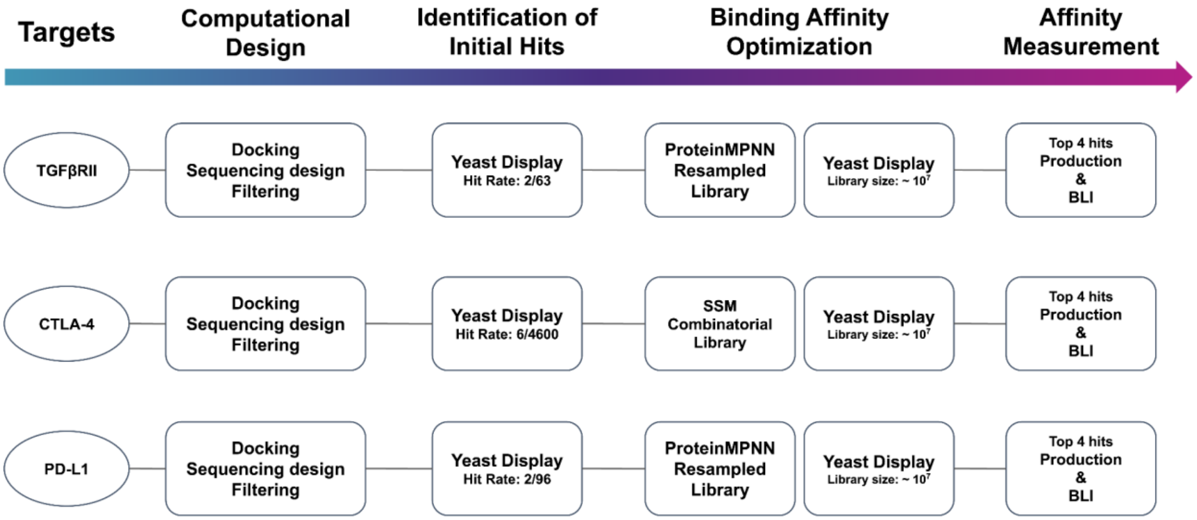

Figure S3. Workflow of Design and Optimization of the 5HCS binders. There are generally four stages for each target including, computational design, identification of initial hits by yeast display (first round of yeast display), binding affinity optimization (second round of yeast display) and final affinity measurement. Library size and hit rates are labeled in the corresponding boxes.

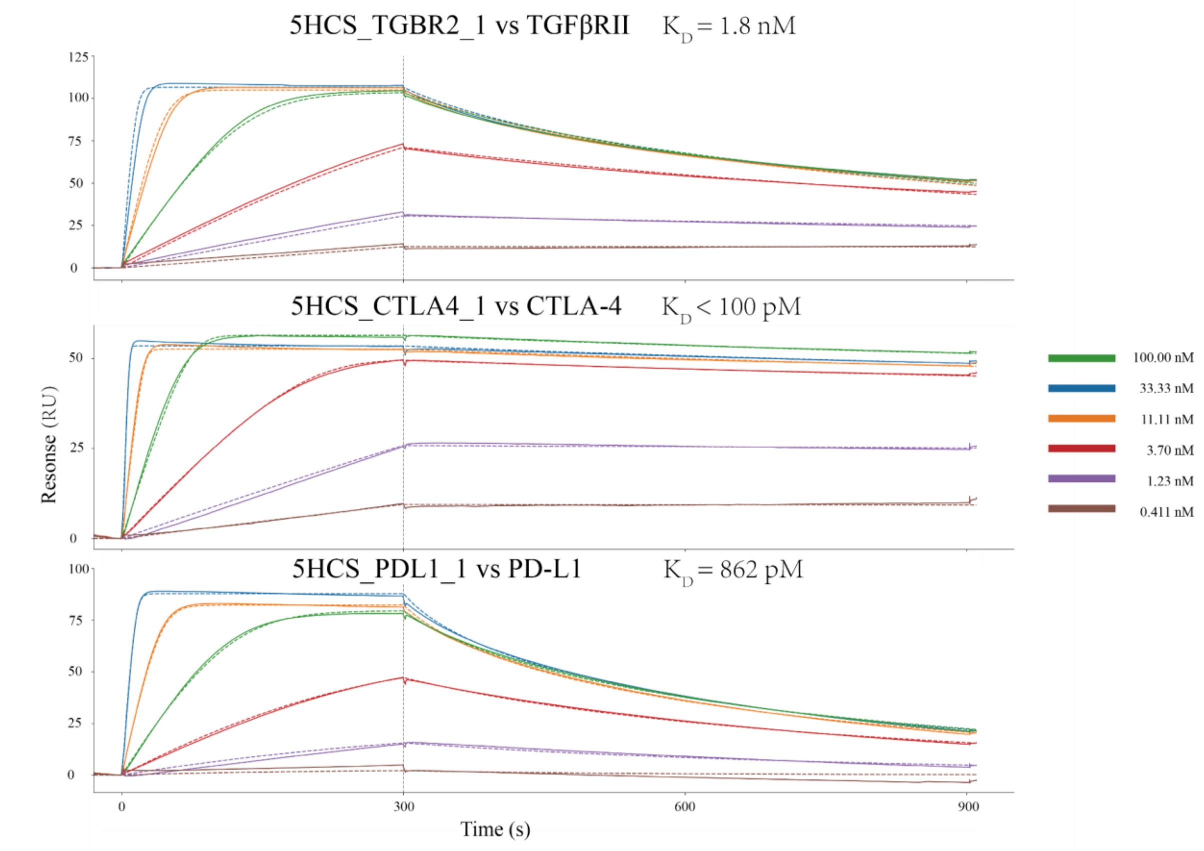

Figure S5. Binding of 5HCS binders to corresponding targets. Binding of 5HCS binder to corresponding targets were measured by Biacore 8K using HEPES running buffer with P20 (0.01 M HEPES pH 7.4, 0.15 M NaCl, 3 mM EDTA, 0.005% v/v Surfactant P20). Target proteins were immobilized on chips(biotin capture chip:TGFβRII, CTLA-4; protein A chip: PD-L1) Binding traces (solid lines) are fitted (dased lines) using Langumuir 1:1 interaction model for kinetic evaluation.

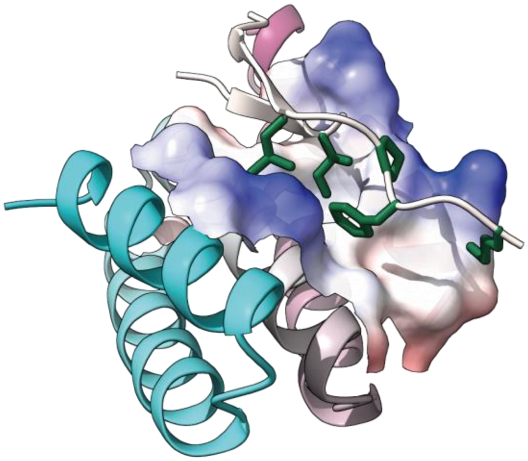

Figure S7. De novo designed binding groove on 5HCS_TGFBR2_1 . 5HCS_TGFBR2_1 shown in cartoon (rainbow) and electrostatic potential surface. TGFβRII binding motif shown as white cartoon and green sticks.

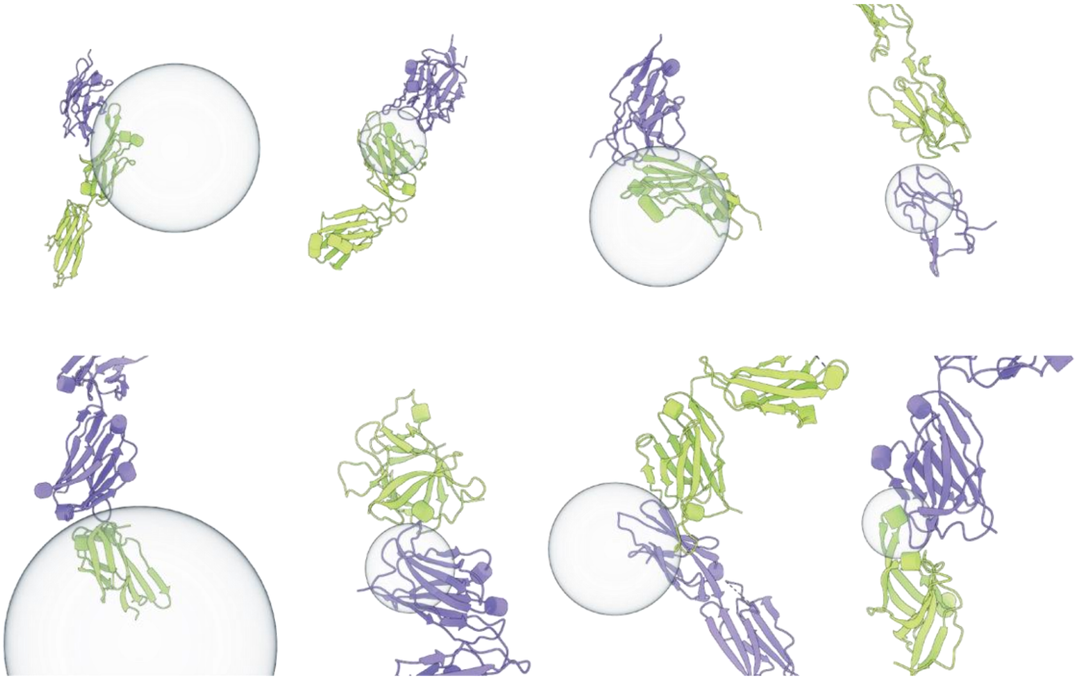

Figure S8. Examples of Immunoglobulin domain head-to-head interactions. PDB ID: 1i8l, 1iqd, 2jjs, 3r08, 3s35, 4g6m, 4hcr, 5d1q. Convex protein surfaces were fitted as spheres.

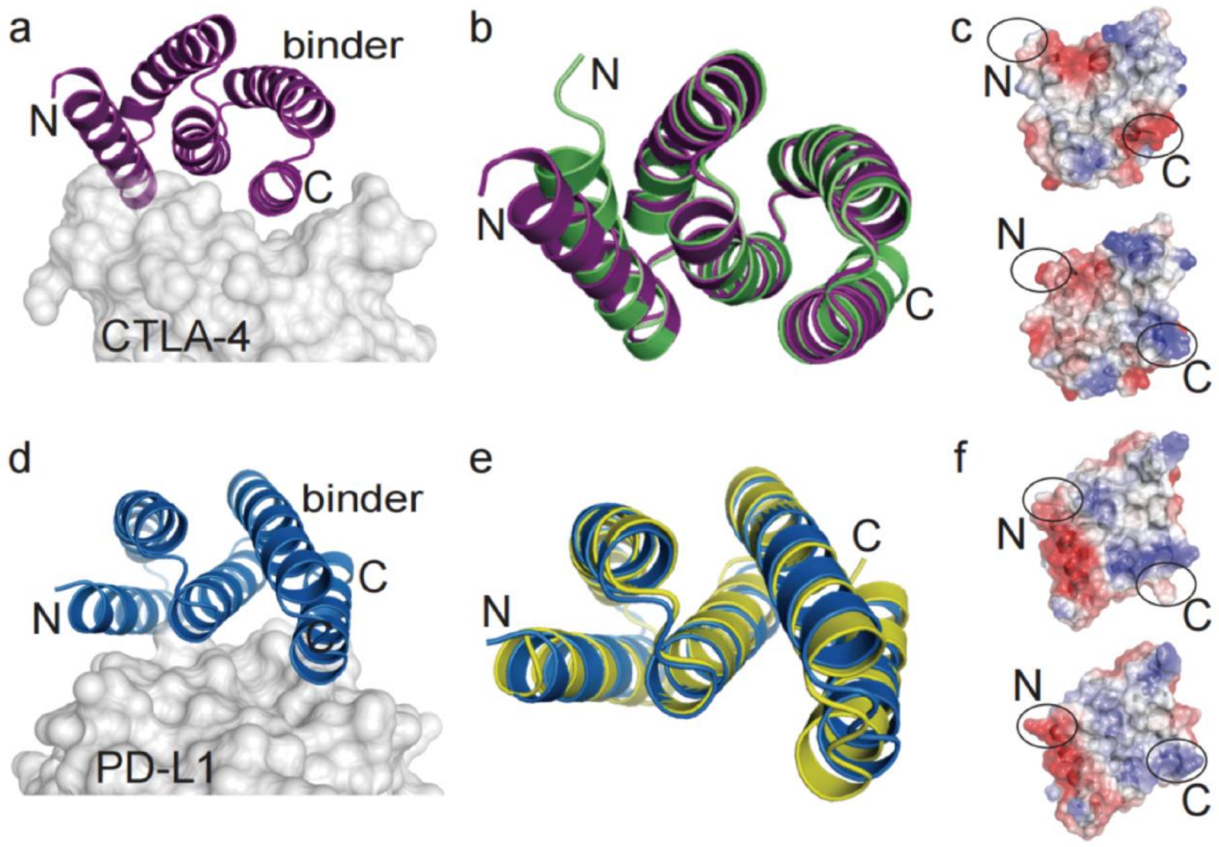

Figure S10. High-resolution structures of 5HCS_CTLA4_2 and 5HCS_PDL1_1.

a, A view of the structure of 5HCS_CTLA4_2 shown in ribbon representation (colored purple). N and C denote the location of the N and C termini. Transparent molecular surface (light gray) depicts modeled CTLA-4 to highlight the binding interface. **b**. Overlay of 5HCS_CTLA4_2 structure to the design model (green). **c**. Comparison of charge distribution at the binder interface between the crystal structure (top) and the model (bottom). **d**. A view of the structure of 5HCS_PDL1_1 (blue, ribbon representation). Part of the modeled PD-L1 is shown as a transparent molecular surface (light gray) **e.** Superimposition of 5HCS_PDL1_1 structure to the design model (yellow). **f.** Electrostatic potential at 5HCS_PDL1_1 interface (top, crystal structure; bottom, de novo model).

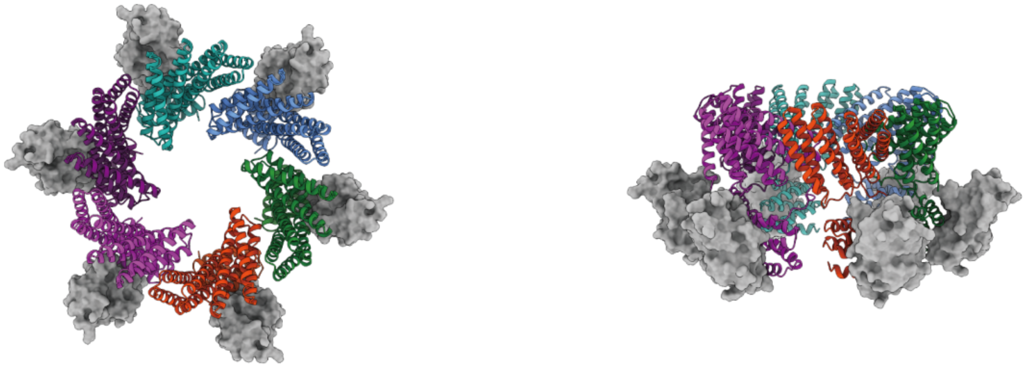

Figure S11. 5HCS_CTLA4_1 fused with hexamers. Top view (left) and side view (right) of the 5HCS_CTLA4_1 fusion with hexamers (cartoon) in complex with CTLA-4 (grey surface). Each chain is colored by a different color.

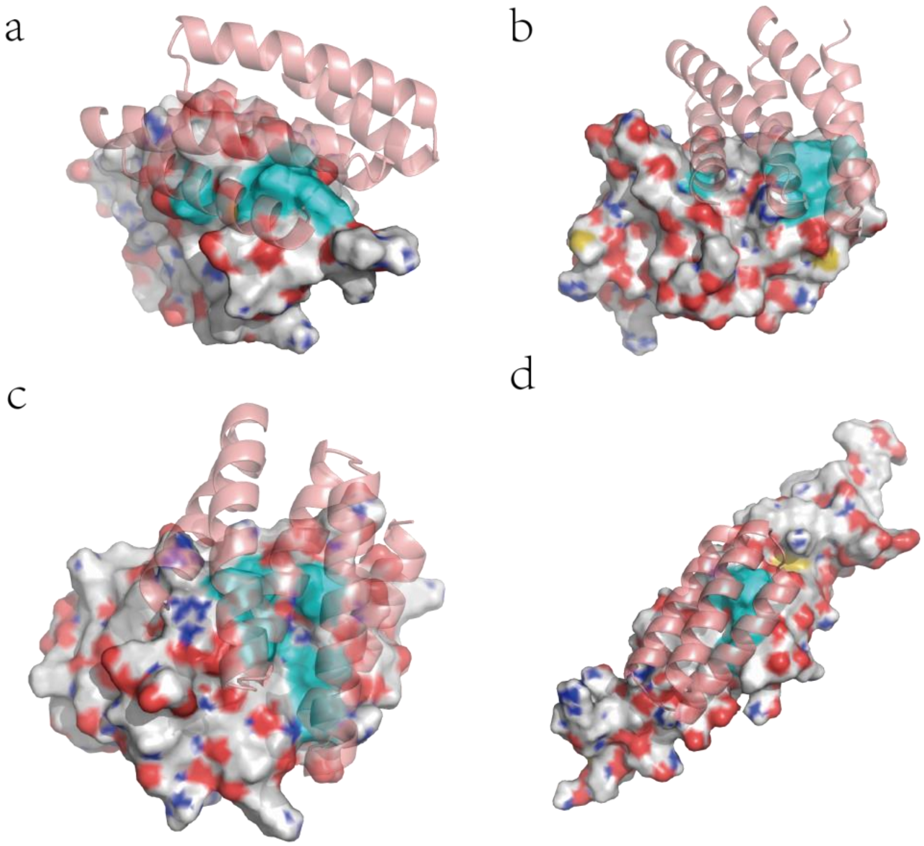

Figure S13. Comparison of binding patches of 5HCS and minibinders.

5HCS_TGFBR2_1 (a), 5HCS_CTLA4_1(b), 5HCS_PDL1_1(b), and Her2 minibinder. Target proteins are shown as surface and binders are shown as cartoons. Key interactions patches are colored in cyan.

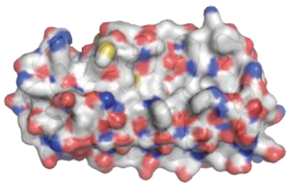

Figure S14. Example of binding surface of Darpin (PDB ID: 8P9E).

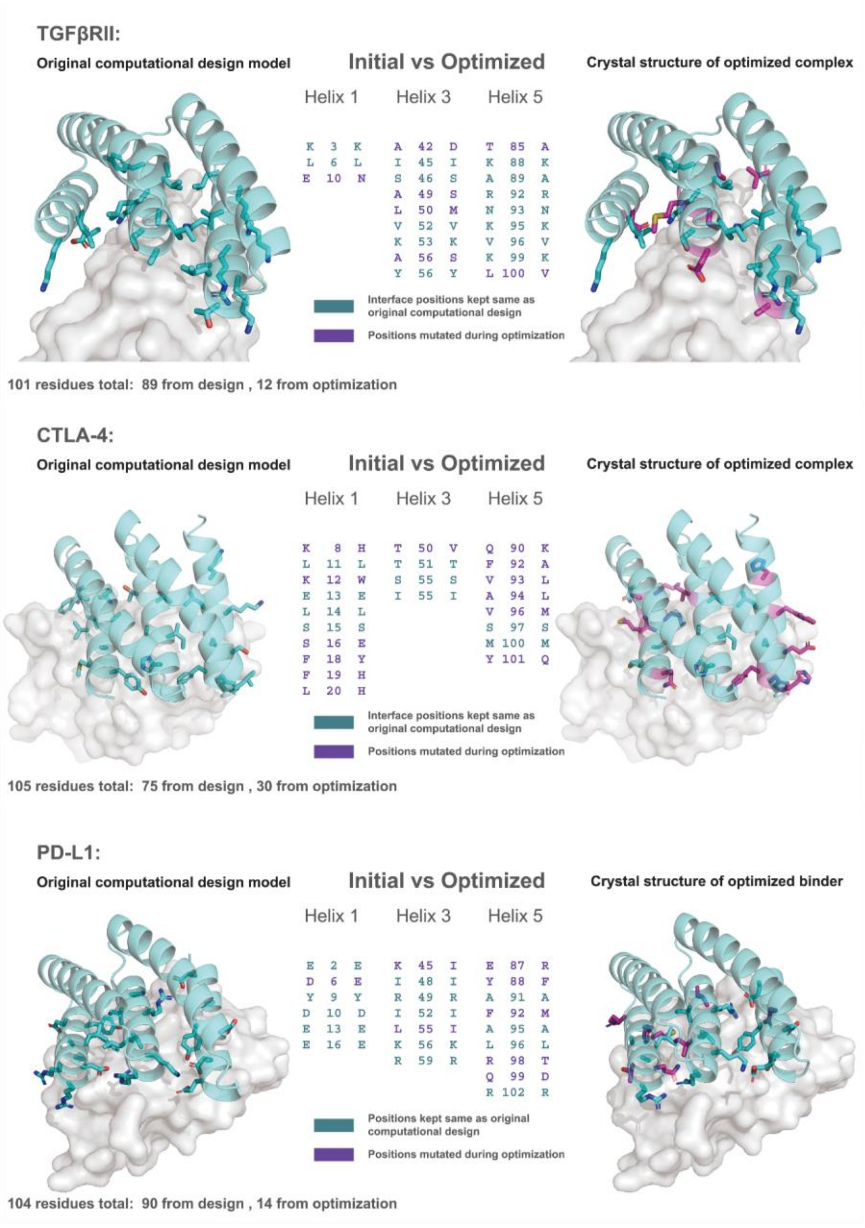

Figure S15. Comparison of original computational design models and crystal structures. The backbones and docking poses of originally computationally designed 5HCS binders for three targets (left) are nearly identical to the crystals of optimized 5HCS binders. A small fraction of residues were optimized through experimental selection methods. Mutations introduced on the interfaces are highlighted in the middle.

Table S1: Interface profiles of de novo designed miniprotein binders

Target	Buried surface area polar/ apolar (Å²)	Convexity binder / target (1/Å)
EGFR	587.5 / 1150.5	3.25e-2 / -2.08e-2
FGFR2	565.34 / 1394.2	-1.5e-3 / -4.6e-2
H3	740.3 / 892.5	1.48e-2 / -3.08e-4
IGF1R	1064.9 / 542.2	7.31e-2 / -8.96e-3
IL7Ra	763.8 / 1039.6	1.70e-2 / 9.46e-3
InsulinR	482.9 / 1101.8	-1.16e-2 / 1.99e-2
PDGFR	687.7 / 1398.0	2.75e-2 / -2.89e-2
TGFb	861.0 / 1026.3	-4.87e-4 / -6.08e-3
Tie2	583.8 / 1032.7	8.05e-4 / 1.06e-2
TrkA	438.2 / 1161.2	5.17e-2 / 1.29e-2
VirB8	413.1 / 1293.4	4.99e-2 / -8.9e-3

Table S2: Crystallization conditions and Crystallographic data statistics

**Protein**	**TGFβRII binder (5HCS_TGBR2_1) complex**	**CTLA-4 binder (5HCS_CTLA4_1) complex**	**CTLA-4 binder (5HCS_CTLA4_2)**	**PD-L1 binder (5HCS_PDL1_1)**
**Ligand$**	**none**	**none**	**none**	**Indole**
**Crystallization condition**	20% (w/v) PEG-MME 5K, 0.4 M $(NH_4)_2$ SO_4, 0.1 M Tris pH 7.4, and 16 – 32 % glycerol	22% (w/v) PEG 3350 and 0.2 M KCl	30 % (w/v) PEG 3350, 0.22 M $MgCl_2$ and 0.0.8 M Bis-Tris pH 5.5 and 0.15 M HEPES pH 7.5	22.5% (w/v) PEG 3350, 0.08 M Bis-Tris pH 5.5 and 2% (v/v) Ethylene glycol
**Cryo preservation**	16 - 32% glycerol	8% (v/v) Ethylene glycol	5% (v/v) Ethylene glycol	8% (v/v) Ethylene glycol
**Data Collection***				
Source	APS 22-ID	BNL 17-ID-2	APS 31-ID	BNL 17-ID-1
Wavelength (Å)	1.00	0.98	0.98	0.92
Number of crystals	1	1	1	1
Space group	$P2_12_12_1$	C2	$P2_12_12_1$	$P4_32_12$
Cell dimensions				
a,b,c (Å)	47.98, 57.17, 78.80	175.75, 33.6,74.47; β = 101.15	39.9, 52.1, 53.5	67.24, 67.24, 101.9
Resolution (Å)	46.28-1.24(1.27-1.24)	20-2.72 (2.82-2.72)	20-1.85 (1.92-1.85)	20-1.88 (1.95-1.88)
Completeness (%)	95.7(53.4)	99.8 (99.3)	99.6 (99.9)	99.8 (99.3)
Total reflections	547697	44220	105488	286708

Unique reflections	61539	11876	9930	19508
Wilson B-factor	14.1	55.07	26.4	36.85
Multiplicity	8.9(2.3)	3.7 (3.6)	10.6 (10.9)	14.7 (14.9)
R_{merge} (%)	5.2(89.8)	8.7 (89.7)	8 (80.3)	5.9 (84.7)
$CC_{1/2}$ (%)	100(42.5)	99.9 (87.2)	99.9 (84.4)	99.9 (89.1)
CC* (%)	100(77.2)	99.6 (87.6)	100 (96.9)	100 (97.1)
<I>/s(I)	18.9(0.6)	10.35 (2.08)	18.4 (3.4)	30.24 (3.66)
**Refinement***				
Resolution (Å)	46.28-1.24	19.5-2.72 (2.82 - 2.72)	19.9-1.85 (2.12-1.85)	19.62-1.88 (1.95-1.88)
Reflections: work/free	61539/3078	11858 (1170)/548 (67)	9928 (985)/430 (45)	19506 (1888)/983 (90)
R_{work}/R_{free} (%)	18.13/19.58	28.7 (32.6)/32.9 (37.4)	19.1 (23.4)/21.8 (26.7)	18.9 (25.7)/21.2 (29.9)
Number of TLS groups	0	0	4	8
Number of atoms				
Protein	3517	3482	2200	1632
Ligand	5	0	4	9
Water	201	0	63	103
Average B-factors ($Å^2$)				

Protein	20.92	50.3	29.1	43.23
Ligand	30.0		39.6	51.75
Water	34.96		38.9	49.93
rmsd.				
Bond lengths (Å)	0.0198	0.004	0.01	0.007
Bond angles (°)	1.9599	0.73	1.1	0.88
MolProbity†				
Favored	98.6% (217 aa)	98% (394 aa)	100% (98 aa)	99.5% (208 aa)
Allowed	100% (220 aa)	100% (402 aa)	none	100% (209 aa)
Outliers	none	none	none	none
Clash score	100th percentile	98th percentile	100th percentile	100th percentile
Molprobity score	100th percentile	98th percentile	100th percentile	100th percentile
**RCSB ID**	8G4K	8GAB	8GAC	8GAD

* Statistics calculated using Phenix (Adam et al., 2010); highest resolution shells indicated in parentheses

† Calculated with the program MolProbity (Chen et al., 2010)

Reference

1. Jumper, J. *et al.* Highly accurate protein structure prediction with AlphaFold. *Nature* **596**, 583–589 (2021).
2. Hiranuma, N. *et al.* Improved protein structure refinement guided by deep learning based accuracy estimation. *Nat. Commun.* **12**, 1340 (2021).
3. Bryan, C. M. *et al.* Computational design of a synthetic PD-1 agonist. *Proc. Natl. Acad. Sci.* **118**, e2102164118 (2021).
